# Prevention and treatment of recurrent Hepatitis B after liver transplantation: the current role of nucleoside and nucleotide analogues

**DOI:** 10.1186/1476-0711-5-8

**Published:** 2006-04-06

**Authors:** Ian R Schreibman, Eugene R Schiff

**Affiliations:** 1 From the Center for Liver Diseases, Division of Hepatology, University of Miami Leonard M. Miller School of Medicine, Miami, FL, USA

## Abstract

The Hepatitis B virus (HBV) is a DNA virus that can cause both acute and chronic liver disease in humans. Approximately 350–400 million people are affected worldwide and up to one million deaths occur annually from cirrhosis and hepatocellular carcinoma. When cirrhosis and liver failure develop, the definitive treatment of choice remains orthotopic liver transplantation (OLT). In the past, an unacceptable HBV recurrence rate with a high rate of graft loss was noted. The use of Hepatitis B immunoglobulin (HBIG) has resulted in improved patient and graft survival rates. The addition of the nucleoside analog Lamivudine (LAM) to HBIG has improved these survival curves to an even greater degree. Prolonged use of LAM will almost invariably lead to the development of viral mutations resistant to the drug. There are now several other nucleoside and nucleotide analogs (Adefovir, Entecavir, Tenofovir, and Truvada) available for the clinician to utilize against these resistant strains. It should be possible to prevent recurrence in most, if not all, post-transplant patients and also to significantly reduce viral loads with normalization of transaminases in those who have developed recurrent infection. The antiviral regimen should be robust and minimize the risk of breakthrough mutations. A prudent approach may be the implication of combination antiviral therapy. This review summarizes the efficacy of previous regimens utilized to prevent and treat recurrent HBV following OLT. Particular attention will be paid to the newer nucleoside and nucleotide analogs and the direction for future strategies to treat HBV in the post transplant setting.

## Introduction

The Hepatitis B virus (HBV) is a DNA virus that can cause both acute and chronic liver disease in humans. Approximately 350–400 million people are affected worldwide and up to one million deaths occur annually from cirrhosis and hepatocellular carcinoma. [1,2] The use of nucleoside analogs has been shown to prevent liver failure as well as prolonging transplant free survival in patients with chronic hepatitis. [3-7] However, if cirrhosis and liver failure develops, the definitive treatment of choice remains orthotopic liver transplantation (OLT). The current estimates suggest that 5–10% of liver transplants performed in the United States are for HBV disease. [8] An unacceptable recurrence rate with an extremely high rate of graft loss was noted initially and HBV infection was actually considered to be a relative contraindication to OLT. [9-11] Fortunately, the use of HBIG resulted in markedly improved patient and graft survival rates. The addition of the nucleoside analog Lamivudine (LAM) to Hepatitis B Immunoglobulin (HBIG) has improved these survival curves to an even greater degree (greater than 80% five year survival rate) while also enabling the treatment team to consider discontinuation of the costly HBIG preparation. [12]

In the pre-transplant setting prolonged use of LAM will almost invariably lead to the development of viral mutations resistant to the drug. Furthermore, prolonged therapy with LAM in the post-transplant setting could also lead to the development of LAM-resistant mutants. Indeed, there have now been several reports of these mutations developing following OLT. [13-22] This raises the question of how to treat these LAM-resistant patients in the post-transplant period. Fortunately, there are other nucleoside and nucleotide analogs (Adefovir, Entecavir, Tenofovir, and Truvada) available now or in the near future for the clinician.

It should be possible to prevent recurrence in most, if not all, post-transplant patients and also to significantly reduce viral loads with normalization of transaminases in those who have developed recurrent infection. The antiviral regimen should be robust and minimize the risk of breakthrough mutations. A prudent approach may be the implication of combination antiviral therapy.

The purpose of this review is to summarize the efficacy of previous regimens utilized to treat recurrent HBV following OLT. Particular attention will be paid to the newer nucleoside and nucleotide analogs and the direction for future strategies to treat HBV in the post transplant setting.

## Prevention of HBV recurrence

### Hepatitis B Immunoglobulin (HBIG)

HBIG first became available for use in 1975. This agent provides a means of passive immunity for the patient. In principle, polyvalent anti-HBs antibodies will bind to and neutralize circulating virions and prevent subsequent graft infection. Anti-HBs also undergoes endocytosis by hepatocytes and binds to HBsAg within cells already infected, thereby decreasing HBsAg secretion. The first large study demonstrating the efficacy of long term HBIG came from the EUROHEP study group in 1993. Three hundred seventy-two patients transplanted for HBV-related liver failure were observed. The risk of HBV recurrence was 75 ± 6% among the 67 patients given no immunoprophylaxis, 74 ± 5 percent among the 83 treated for two months, and 36 ± 4 percent among the 209 treated for six months or longer (P < 0.001). Improved patient survival (75 versus 45 percent) at three years was also noted among those patients receiving passive prophylaxis with HBIG. Multivariate analysis revealed that long-term administration of HBIG was associated with a relative risk reduction of 3.3 for the development of recurrent HBV. [23] These findings have now been confirmed in multiple studies and the median rate of recurrent HBV in patients receiving long-term HBIG is approximately 20% over one to two years. [24-33]

There are several drawbacks to the use of HBIG. First is its cost. Most regimens currently in use in the United States range from $80,000 to $200,000 for the first year. These costs also include the cumbersome IV infusion sets and monitoring of the patient during administration. [29] The supply of HBIG is limited. Significant side effects have been noted with the infusion including headaches, flushing and chest pain. [24]. Lastly, the development of escape mutants can be seen reducing the efficacy of HBIG. These escape mutants are typically due to mutation in the HBsAg at the "a" determinant loop. [34] The trend has been toward lower doses given intramuscularly.

In the past, the use of monoclonal antibody to the HBV surface antigen led to an unacceptable rate of breakthrough infections. [35,36] However, a recent study by Galun et. al. [37] showed promising results using a mixture of two monoclonal antibodies in a phase I clinical study. Patients developed a rapid and significant decrease in HBV-DNA levels. Future studies are warranted, but this preliminary data suggests that monoclonal antibody preparations could replace the current polyclonal HBIG.

### Lamivudine (LAM)

Lamivudine (LAM) was the first nucleoside analog to be approved for the use of chronic HBV. Its mechanism of action is the inhibition of the DNA polymerase of the virus and suppression of HBV replication. [38,39] LAM has also been shown to be safe in patients with decompensated liver disease; it is well tolerated and achieves a rapid loss of HBV DNA in the serum. LAM is preferred over the use of the Interferons which are contraindicated in patients with advanced cirrhosis. [3-7]

In the pre-transplant setting, the drug has been shown to be potent. Undetectable HBV DNA can be achieved in most patients within 2–3 months. [3,6,7,13,40-47]. Furthermore, histologic improvement may be seen in 49–56% of patients after receiving one year of therapy. [34,48-51] Yao showed that the use of LAM could also improve the Child-Pugh-Turcotte (CPT) score by more than or equal to three points in 60% of patients receiving the drug and even reduce the necessity for OLTx. [7]

The major drawback to the use of LAM is the development of resistance to the drug caused by mutations in the reverse transcriptase gene. The most common mutations occur in Domain C of the HBV polymerase at the tyrosine-methionine-aspartate-aspartate (YMDD) locus. Other mutations can occur at Domain B in conjunction with the YMDD mutations. [52] Liaw showed that the cumulative rates of LAM resistance were 14%, 38%, 49%, 66% and 69%, one, two, three, four, and five years after initial therapy respectively. [53] Other studies have showed similar rates of resistance with resistance rates of 24% and 70% after one and four years of therapy respectively. [50,51,54]

In an attempt to lower the cost, and avoid the side effects and cumbersome administration of HBIG, multiple studies have examined the use of LAM prior to and following transplant to prevent HBV recurrence. Perillo et. al. conducted the largest multicenter study in North America in which 77 liver transplant candidates were treated with LAM (100 mg daily) without the adjunctive use of HBIG. Treatment was initiated prior to and after transplantation. Forty-seven eventually underwent liver transplantation. Re-infection with HBV occurred at a rate of 40% over the subsequent three years and HBV-DNA polymerase mutants were detected in 15 (21%) of the transplanted patients. [6] Other studies with LAM monotherapy have also been disappointing with high rates of HBV recurrence ranging from 23–50% of patients. [41,43,55-57] The high rates of recurrence were due to the expected emergence of escape mutations in the YMDD locus. For this reason, LAM prophylactic monotherapy has been abandoned in favor of combination therapy with HBIG.

### Lamivudine and Hepatitis B Immunoglobulin combination therapy

In contrast to monotherapy, LAM in combination with HBIG has been potent in preventing the recurrence of HBV in the post-transplant setting. Indeed, following the results of several studies, the standard of care has become administration of LAM in the pre-transplant setting (ideally, at least four weeks prior to transplantation) followed by the combination of LAM and HBIG in the post-transplant setting. [58] HBV recurrence rates are usually less than 10% one to two years following transplantation. Furthermore, HBV DNA levels by PCR are also typically undetectable. [13,40,42,59-66]

These favorable response rates are likely secondary to the additive effects of LAM with the HBIG. Another added benefit of combination therapy is that HBIG can be administered in reduced amounts thus reducing costs and increasing availability of HBIG. [13,40] Some have argued that HBIG could even be stopped at some point after transplantation. This issue represents an impending controversy that requires further investigation before a robust recommendation can be made, although our center often discontinues HBIG at some point following OLT depending upon the individual patient, serologic studies and viral load.

### Adefovir (ADV)

It is now well known that a significant number of patients can develop LAM resistant strains of HBV while receiving this drug in the pre-transplant setting. Furthermore, an increasing number of patients may become primarily infected with a LAM resistant HBV mutant. In this setting, the use of LAM for prevention of recurrent HBV in the post transplant setting is of limited value. Adefovir dipivoxil (ADV) is an orally available prodrug of Adefovir- a nucleotide analog of adenosine monophosphate that has been shown to have activity against both wild type and LAM-resistant HBV. Improvement in histology, LFT's and viral DNA levels have been seen with the use of this drug. [67-69]. The drug is very well tolerated and the only significant side effect is risk of increased nephrotoxicity after 20 or more weeks of use. [70]

Several large studies have shown the utility of ADV in the pre and post liver transplantation setting. The largest study by Schiff et. al. showed that among patients with LAM-resistant HBV and who were pre-OLT, 81% achieved undetectable serum HBV DNA. Furthermore, serum ALT, Albumin, bilirubin and prothrombin time normalized in 76%, 81%, 50% and 83% of these pre-OLT patients respectively. Furthermore, the Child-Pugh-Turcotte score improved in over 90% of cohorts. Unlike the use of LAM, no resistance to ADV was identified after 48 weeks of therapy in this population. [71] Many patients came off the transplant list because of reversal of the decompensated state.

Another study describing the use of prophylactic ADV was from Lo et. al. They describe 16 patients who had developed YMDD mutations while on the waiting list for OLT. Eleven patients received ADV for a median of 20 days (range 8–271 days) before transplantation while 5 patients started the drug at the time of OLT. The median follow-up period after OLT was 21.1 months (range 4.4–68.9 months). One patient died of a cause unrelated to HBV 12.2 months after transplantation. Fifteen patients (94%) were alive with the original graft. Pre-OLT HBV DNA levels at the time of breakthrough were available in 15 patients ranging from 2 × 10^3 ^– 4.69 × 10^9 ^copies/mL (median 1.42 × 10^7^). Lo's cohort was divided in half: eight patients received HBIG (in addition to ADV and LAM) for a median of 24 months whereas the other 8 patients received prophylaxis with ADV and LAM alone. All 16 patients cleared the HBV DNA and had no evidence of recurrence; furthermore, all remained HBeAg negative. The graft survival was 94% at a median follow up of 21 months. Lo concludes that add-on ADV plus LAM should be the "preferred approach in those patients who have already developed resistance to lamivudine so as to avoid the emergence of multiresistant viral strains." [72]

Previous studies reported a low rate of ADV resistance- occurring in less than 2% of patients after 96 weeks. [66,68,73,74] However, the emergence of ADV resistant HBV mutants is now considered to be clinically significant over time when given as monotherapy; resistance has been reported in 18% of patients after 4 years of therapy and 22% after 2 years of therapy. [75-77].

It is expected that the development of ADV mutations will similarly become a problem in the post-transplant setting. Villenueve recently reported the first case of a patient who developed sequential selection of LAM and ADV resistant strains of HBV in a liver transplantation patient. [20] The patient was a 52 year old Cambodian who was initially HBV DNA negative and HBsAg positive. He was given prophylactic HBIG alone in the peritransplant setting without any nucleoside analogs. Virologic breakthrough with LAM resistance mutations in L180M and M204V followed 20 months later. ADV was added to LAM and after reduction in DNA levels, LAM was discontinued and ADV was continued alone. A subsequent reversion to wild type took place. A second virologic breakthrough occurred after nearly two years of ADV monotherapy with the selection of the resistance mutation N236T of the D domain of the HBV polymerase. [20] Xiong et. al. had recently described this mutation in 2/124 patients receiving chronic ADV therapy for two years. [74] LAM was reintroduced with ADV after which the patient developed undetectable levels of the virus.

Both the Schiff and Lo study suggest that ADV provides safe and effective prophylaxis in those patients with LAM resistant HBV infection.

An area for future investigation is whether the use of ADV in combination with LAM should be utilized. There are several potential benefits with combination therapy. The chance for breakthrough mutations to either LAM or ADV is significantly less when the agents are used in combination. [72,77,78] Snow et. al. observed a patient population of 467 patients with LAM-resistant HBV. He reported that resistance to ADV was only seen in those who stopped LAM. [78] Fung et. al. studied all HBV patients in a tertiary care center receiving ADV. He found that none of the patients receiving combination therapy (LAM + ADV) developed ADV resistance; among those who had switched to ADV monotherapy after combination therapy, 5 of 18 patients (28%) developed resistance. [77]

Another potential benefit of combination therapy would be to eliminate the need for HBIG with its economic cost and potential toxic side effects (see discussion above). Note that in Lo's study, all eight patients who did not receive HBIG remained alive with normal chemistries at a median follow up of 15.1 months (range 4.4– 26). Furthermore, there was no histologic evidence of recurrence. Six of these eight had spontaneous HBsAg seroconversion. Although, two of the eight remained HBsAg positive, both had undetectable DNA levels with normal LFT's. The possibility of a viral breakthrough with resistance to both LAM and ADV should be low given the relatively low frequency of ADV resistance and lack of cross-resistance to LAM when given in combination. [72] The duration of HBIG therapy remains a contentious issue; to suggest its elimination altogether is controversial and worthy of further investigation.

## Treatment of HBV recurrence

### Lamivudine

Prior to combination HBIG/LAM, graft infection was frequently seen. As described above, this was due to the high recurrence rates in patients who received HBIG or LAM as monotherapy alone or in those who received no prophylaxis at all. De Novo HBV is also seen in the post transplant setting. Fortunately, LAM is very effective in treating HBV infection of the graft and remains the first line agent. Perillo performed a large multicenter center of 52 HBV-DNA positive patients. After one year of treatment, LAM resulted in a 68% loss of HBV serum DNA with an 11% HBeAg seroconversion. [79] Other studies have shown similar improvements with HBV-DNA negativity ranging from 75 to 100%, although HBeAg serum conversion is generally less than 30%. [59,80,81] This low rate of serum conversion is consistent with prior reports in the non-transplanted population. [67]

Unfortunately, breakthrough is seen just as in the non-transplanted population in up to 50% of patients within the first one to two years following transplantation [59,79-81]. It should be expected that with even longer follow up the development of LAM resistant mutants should approach 70%. Continuation of LAM for graft infection that develops while receiving prophylaxis with LAM ± HBIG is not indicated. Furthermore, the use of LAM as a primary treatment agent is ineffective for de novo graft infection with a LAM resistant mutant. In these scenarios, ADV should be initiated, although Tenofovir or Truvada may ultimately replace ADV pending future studies.

### Adefovir

The beneficial effects of ADV in the chronic HBV population suggested that ADV may be helpful for the development of LAM-resistant mutants in the transplanted population receiving prophylactic combination therapy or in the development of de novo LAM-resistant HBV infection. Schiff et. al. performed a large multicenter study investigating the utility of Adefovir examined two cohorts with LAM-resistant HBV infection. The beneficial results seen in the pre-OLT cohort described above (see Prevention Section) were also found in the post-OLT population. One hundred ninety-six patients were followed for a median of 56.1 weeks. After 24 and 48 weeks of therapy with ADV, a 3.1 and 3.4 drop in log PCR DNA levels was seen. In addition, 34% achieved undetectable serum HBV DNA levels. Serum ALT, albumin, bilirubin and prothrombin time normalized in 49%, 76%, 75% and 20% of these patients respectively. A 93% one year survival was seen. [71] Several studies and case reports have confirmed the efficacy of ADV in the setting LAM-resistant graft infection whether it be the development of a mutant while on prophylactic therapy or de novo LAM-resistant HBV graft infection. (See Table 1.) Multiple studies have shown that there is a low rate of HBeAg seroconversion when Adefovir is used for patients with chronic Hepatitis B. [67] This observation is also found in the post transplant infected patients.

**Table 1:** The use of Adefovir for the treatment of HBV graft infection

Author (Reference)	No of Patients	No patients DNA Pos	No patients HBeAg Pos	Treatment duration	DNA Negative following Treatment	HBeAG Seroconversion	DNA Change Median	Development of Adefovir Resistant Mutants
Echanojauregui [82]	7†	7	5	48 weeks	3	1	-3.19	None reported
Schiff [71]	196*	172	Not reported	56.1	34	Not reported	-4.3	None reported
Wai [16]	4*	4	Not reported	24 months (one only received 9 months of therapy)	0	Not reported	-2.0	None reported
Neff [19]	9*	9	7	6– 48 months Median 30 months	0*	4	Not reported*	None reported
Toniutto [22]	1†	1	0	2 months	1	N/A	Initial load not reported but became undetectable	No
Beckebaum [83]	1*	1	1	15 months	0	0	2.45 billion to 32, 100	No
Akay [17]	2*	2	Not reported	4 months	2	Not reported	2000 and 17 pg/mL respectively to undetectable	No

*-Cases were the development of LAM-resistant mutants while on prophylactic therapy

†- Cases were from the development of de novo LAM-resistant HBV graft infection

ADV is thus both an effective and safe drug to treat graft infection and prevent clinical deterioration of patients affected with the development LAM resistant HBV graft de novo infection or for LAM-resistant breakthrough mutants. (See Table 1)

Neff's study [19] described a total of 14 patients who had the development of LAM resistant mutants. Nine patients were switched from LAM to ADV. The other 5 patients were treated with Tenofovir. Of the patients given ADV, one died during follow-up. Of the 8 alive, all but one had reductions in HBV DNA.

Toniutto's study [22] describes one patient who developed de novo HBV graft infection. 3 months after treatment of LAM, LAM resistance developed. The patient was given two months of ADV plus LAM with HBsAg seroconversion and undetectable HBV DNA levels. Therapy was stopped thereafter and the patient remains HBV negative 13 months later.

None of these studies and case reports described any significant nephrotoxicity with the use of ADV. In post-OLT patients, there are multiple factors, in particular the use of calcineurin inhibitors, that contribute to renal dysfunction. Some patients did develop a rise in serum creatinine which was easily treated with reduced frequency dosing of ADV.

### Entecavir and Tenofovir

Although ADV is generally well tolerated, there does exist a risk of nephrotoxicity with this agent. Furthermore, as described above, there exists the possibility of N263T and other ADV resistant mutants. In these cases, it may be desirable to use either Tenofovir Disoproxil Fumarate or Entecavir.

Entecavir is a carboxylic analogue of guanosine and is the most recently FDA approved drug for the treatment of chronic Hepatitis B. Like Adefovir, it has been shown to be a potent antiviral agent for the wild type and LAM-resistant forms of chronic HBV. [84-86] It is expected that Entecavir will be efficacious in the prevention and treatment of recurrent HBV following liver transplantation. The first reports of Entecavir's use in this setting are now being reported. [87]

Tenofovir is currently only approved for use in HIV positive patients but has been to be effective for LAM resistant mutants and for those patients who have failed prior ADV and LAM. It is a nucleotide analogue and acts as a reverse transcriptase inhibitor. [88-90] Several case reports have now been reported in which Tenofovir produced a well tolerated, successful antiviral response in patients before and after OLT.

Taltavull describe a 54 year old male who developed YMDD mutations while receiving LAM therapy. ADV produced a suboptimal response: DNA levels were still positive after 8 months of therapy. His clinical status continued to decline with spontaneous bacterial peritonitis and worsening of CPT status. Tenofovir was then added and produced a rapid dramatic decline in DNA levels to undetectable levels 4 weeks after treatment. He continued on this treatment up to OLT after which time he received only HBIG, LAM and ADV. [91] The patient's outcome remained excellent 21 months following OLT.

In 2004, Neff et. al. [21] reported the successful use of Tenofovir in patients who developed the Lam resistance following OLT. From June, 1998 through December, 2003, 25 patients transplanted for HBV were managed on chronic LAM therapy. Sixteen patients (64%) developed resistance to LAM between 10–85 months (median 26) following OLT. Eight of these patients were administered Tenofovir at a dose of 300 mg/day 1–66 months after the development of resistance. Therapy was continued for 14–26 (median 19.3) months. All 8 patients experienced DNA suppression with 7 having undetectable viral loads. Creatinine clearance was not impaired nor was any other adverse event reported. Both the Taltavull and Neff study suggest that Tenofovir is a safe and effective therapy for those patients who develop LAM-resistant mutants after OLT. It also stands to reason that the use of Truvada (Emtricitabine and Tenofovir) will also be useful in this setting; although, like Tenofovir, it is not yet FDA approved for this use.

### Conclusion

The Hepatitis B virus is a DNA virus that can cause both acute and chronic liver disease in humans. If cirrhosis and liver failure develops, the definitive treatment of choice remains orthotopic liver transplantation (OLT). In the past, recurrence of HBV was common following OLT- leading to unacceptable rates of graft loss and increased morbidity and mortality.

With the advent of new antiviral therapy, it should be possible to prevent recurrence in most, if not all, post-transplant patients. Ideally, the antiviral regimen should be robust and prevent breakthrough mutations. In decompensated cirrhotic patients, the use of combination therapy has the advantage of reducing the risk of escape mutations. A prudent approach in preventing recurrence may be the implication of combination antiviral therapy in the post-transplant setting as well. This remains an unanswered question. As discussed above, most studies using combination therapy have been small; although favorable results are seen.

Fortunately, for those who have already developed recurrent disease, newer agents, either alone or in combination, are able to achieve significant reductions in the HBV DNA level and normalization of transaminases.

A pending controversy is the duration of HBIG therapy. While the use of HBIG infusions is universally accepted as advantageous in the perioperative setting, the use of combination oral agents may make it possible to stop this costly therapy after a short period of time or avoid it entirely. This remains a contentious issue that requires further studies before a definitive recommendation can be made.
